# Do students from public schools fare better in medical school than their colleagues from private schools? If so, what can we learn from this?

**DOI:** 10.1186/s12909-018-1162-x

**Published:** 2018-03-27

**Authors:** Cristina Costa-Santos, Pedro Vieira-Marques, Altamiro Costa-Pereira, Maria Amélia Ferreira, Alberto Freitas

**Affiliations:** 1 MEDCIDS – Departamento de Medicina da Comunidade, Informação e Decisão em Saúde, Faculdade de Medicina da Universidade do Porto, Rua Dr. Plácido da Costa, s/n, 4200 – 450 Porto, Portugal; 2 CINTESIS – Center for Health Technology and Services Research, Faculdade de Medicina da Universidade do Porto, Porto, Portugal; 3 Serviço de Informática, Faculdade de Medicina da Universidade do Porto, Rua Dr. Plácido da Costa, s/n, 4200 – 450 Porto, Portugal; 4 Departamento de Ciências da Saúde Pública e Forenses e Educação Médica, Faculdade de Medicina da Universidade do Porto, Rua Dr. Plácido da Costa, s/n, 4200 – 450 Porto, Portugal

**Keywords:** Performance in medical school, Access to medical school, Grade inflation, Public and private secondary schooling

## Abstract

**Background:**

Internal grade inflation is a documented practice in secondary schools (mostly in private schools) that jeopardises fairness with regard to access to medical school. However, it is frequently assumed that the higher internal grades are in fact justifiable, as they correspond to better preparation of students in private schools in areas that national exams do not cover but nevertheless are important. Consequently, it is expected that students from private schools will succeed better in medical school than their colleagues, or at least not perform worse. We aimed to study whether students from private schools do fare better in medical school than their colleagues from public schools, even after adjusting for internal grade inflation.

**Methods:**

We analysed all students that entered into a medical course from 2007 to 2014. A linear regression was performed using mean grades for the 1st-year curse units (CU) of the medical school curriculum as a dependent variable and student gender, the nature of students’ secondary school (public/private), and whether their secondary school highly inflated grades as independent variables.

A logistic regression was also performed, modelling whether or not students failed at least one CU exam during the 1st year of medical school as a function of the aforementioned independent variables.

**Results:**

Of the 1709 students analysed, 55% came from public secondary schools. Private (vs. public) secondary school (β = − 0.459, *p* < 0.001) and whether secondary schools highly inflated grades (β = − 0.246, *p* = 0.003) were independent factors that significantly influenced grades during the first year of medical school. Having attended a private secondary school also significantly increased the odds of a student having failed at least one CU exam during the 1st year of medical school (OR = 1.33), even after adjusting for whether or not the secondary school used highly inflated grades.

**Conclusions:**

It is important to further discuss what we can learn from the fact that students from public secondary schools seem to be better prepared for medical school teaching methodologies than their colleagues from private ones and the implications for the selection process.

## Background

Unfairness regarding access to higher education and, in particular, medical school, has been debated worldwide [1–6]. Moreover, achievement, as determined by different selection methods used by medical schools, may differentially predict performance at the various stages of medical education and clinical practice [7].

When higher education application scores include internal scores obtained in secondary education, private secondary schools, which are dependent on the fees that they charge students and are more likely to be under market pressure, may have more incentive to inflate internal grades [8]. Consequently, students from a higher socio-economic status background may have an advantage with regard to access to medical school.

The Portuguese higher education admission process uses the *numerus clausus* method to manage students’ applications to available programmes. Each student may apply to a maximum of six programmes – the courses and number of places available in each programme are defined by the Universities and later approved by the Ministry of Education. The application score is a weighted average of the internal scores obtained in upper secondary education (equal or above 50%) and the scores obtained in national exams. National exams are required not only for accessing higher education but also for the completion of secondary education. National examinations are the same across the country and are scored by anonymous external evaluators. Because internal scores obtained in upper secondary education have a weight equal to or greater than 50% of the application score, students who plan to apply to a very competitive programme, such as medical school, tend to strategically choose, three years before the application, secondary schools where grades are usually inflated. Usually, the more common choice is a private school, because, although there are public schools that also inflate students’ scores, this inflation occurs mostly in private schools [5]. Therefore, students frequently aspire to attend private schools, even when high fees are charged, over public schools, where no fee is charged.

Consequently, students that are able to pay these fees may benefit from an unfair advantage, the inflation of internal grades, when competing for scarce positions at medical schools. Although Portuguese public higher education is commonly regarded as the most prestigious with high international rankings, at the secondary level, parents and students commonly prefer private schools. In fact, the notion that private secondary schools benefit their students through grade inflation coexists with the rumour that private schools also have higher teaching standards [5]. The Portuguese government, which is concerned with this problem, reports annually through an on-line portal statistical information on demographics and the academic performance of students enrolled in scientific-humanities programmes in secondary education in Portugal [9]. Amongst other data, this portal provides an indicator of the alignment of internal grades awarded by each secondary school with marks awarded by all of the other secondary schools to students with similar national exam results [9].

For example, if the internal grades assigned by School A are systematically higher than the internal grades assigned by School B to students who ultimately achieve the same results on the national exams, then it is possible that the secondary School A uses considerably different evaluation criteria for student performance (less demanding) than School B. Through this methodology, all secondary schools are classified as being either highly misaligned up (high internal grade inflation), misaligned up (internal grade inflation), aligned, misaligned down (internal grade deflation) or highly misaligned down (high internal grade deflation) [9].

The problem of inequality with regard to access to higher education is not only a concern in Portugal. In fact, some French and English researchers have claimed that grades are awarded according to the characteristics of the student population at a given school, without a clear link to the actual value of each student, thus posing obvious implications for the equality of opportunities [10, 11]. In the particular case of medical school, according to an Australian study, admission scores are positively associated with prior secondary school education in a fee-paying independent school [12]. Another study performed in the United Kingdom suggests that private schooling improves the likelihood of gaining admission to medical school [13]. According to this study, academic excellence in secondary school does not make for a certain pathway into medical school. In fact, applying with good grades after attending private school is a more certain pathway [13]. Recently, a study about Portuguese public and private secondary schools showed that private schools inflate their students’ scores in comparison to other types of schools. As a consequence, students from private schools benefit from an unfair admission process, one that improves their chances of accessing higher education, particularly medical school (the most competitive programme) [5]. A report from Porto University rectory characterises the 4313 students who were admitted in 2009 to undergraduate programmes at the University of Porto and describes their situation one and three years following admission [14]. According to this report, students who came from public secondary schools had a greater presence in the top 10% of students than those who came from private schools [14]. According to the same study, the difference between public and private secondary education (e.g., better performance in university for students who came from public secondary schools) was emphasised in medical school [14]. This tendency (better performance in university for students who came from public secondary schools) can be found in other countries. In fact, a Norwegian study showed that students who came from secondary private schools had a lower level of academic performance in medical school than other students [15]. Another study aimed at finding determinants of performance in United Kingdom Universities revealed that the performance was significantly lower for students who attended a private school prior to university [16].

Private schools (with fees) in Portugal disproportionately increase their students’ grades (data from Portuguese government, on-line portal with national data) [9].

Despite this evidence, the argument that higher internal grades are in fact justifiable because they correspond to better preparation of students in private schools in areas that national exams do not cover but nevertheless are important for academic success, which is frequently assumed to be true, cannot be completely dismissed. If this is indeed true, then it should be expected that students from private schools will succeed better at medical school than their colleagues from public schools, or at least not perform worse.

Consequently, our aim was to determine whether students from private schools performed better in medical school, or at least not worse than their colleagues from public schools, even after adjusting for internal grade inflation.

## Methods

The data for this study correspond to the population of a medical school over an eight-year period. Specifically, data were collected at the end of the 2014/2015 curricular year (July 2015) from the University of Porto administrative database, including all students from the general contingent who were admitted to the Faculty of Medicine at the University of Porto (FMUP) between the 2007 and 2014 in the first phase of admission.

Consequently, grades from at least one year at FMUP (1st curricular year) were collected for all students. For students who were admitted to FMUP in 2007, 2008 and 2009, we collected grades for all six curricular years. For students who were admitted to FMUP in 2010, 2011, 2012, 2013 and 2014, we collected grades for the first five curricular years, first four curricular years, first three curricular years, first two curricular years and first curricular year, respectively.

We excluded students who transferred from other programmes and those who finished the first year by accreditation based on previous academic performance. Students who re-entered and those who completed secondary education in non-Portuguese schools were also excluded.

Students generally prefer FMUP over other medical schools. Consequently, the last student accepted to FMUP had, in recent years, always had a better application score than the last students placed in other Portuguese medical schools.

For each student, we averaged their scores obtained during a curricular year whenever the student attempted to complete a Course Unit (CU) and earn approval. An exam failure was designated when a student was evaluated (in a final exam of a CU) without success. For each student, we counted the number of exam failures in each curricular year. As there were not many students with more than one failure in each curricular year, we dichotomised this variable into students with at least one failure in a CU exam and students without any failures.

We accessed information provided by the Ministry of Education and Science for each secondary school [9] from 2009 to 2014 to determine whether internal grades assigned by the represented schools were highly misaligned (high internal grade inflation). High internal grade inflation (misalignment) means that the internal scores assigned by a particular school are, on average, higher than those assigned by all of the other schools in the country to students with similar scores on the national exams.

It was not possible to access this information regarding internal grade alignment before 2009. Consequently, the analysis comparing students who came from private and public schools encompassed an 8-year period (from 2007 to 2014). However, the analysis comparing students who came from schools that inflate internal grades to those whose schools did not encompassed a 6-year period (from 2009 to 2014).

Multiple linear regression was carried using the mean grades for first-year CU as a dependent variable and gender, type of secondary school (public/private), and whether the secondary school used highly inflated grades during the year in which the student applied to medical school as independent variables.

Logistic regression was also performed to model whether or not students failed at least one exam during the first year of medical school as a function of the same above-listed independent variables.

## Results

Between 2007 and 2009, a total of 1709 students entered the medical school in the first phase of admission. Of these, 1068 (62%) were female and 939 (55%) came from a public secondary school. For three students, we could not obtain information about the school where they completed their secondary education.

Six students cancelled their enrolment in medical school before any attempt to complete a CU. For each of the remaining 1703 students, we averaged their grades obtained when they tried to earn a CU and succeeded. Students for whom it was not possible to obtain information about their secondary school of origin were removed. Thus, 1700 students were retained in the analyses.

Table 1 presents mean grades (students’ grades range from 0 to 20) obtained in medical school for students who came from public and private secondary schools for each curricular year. Only students who earned at least one CU in the respective curricular year were considered.

**Table 1:** Mean grades obtained in medical school from students who came from public and private secondary schools for each academic year

Curricular year	Private secondary school (A)		Public secondary school (B)		Mean difference	Cohen’s d	*p*-value
	N	Mean (SD)	N	Mean (SD)	(A-B)		
1	767	12.8 (1.2)	933	13.4 (1.3)	−0.6	0.48	< 0.001
2	655	13.7 (1.1)	823	14.2 (1.3)	−0.5	0.42	< 0.001
3	550	13.7 (1.3)	713	14.1 (1.4)	−0.4	0.30	< 0.001
4	435	15.1 (1.2)	616	15.4 (1.2)	−0.3	0.25	< 0.001
5	347	16.1 (1.1)	492	16.3 (1.0)	−0.2	0.19	0.003
6	238	17.5 (0.7)	356	17.4 (0.7)	0.1	0.14	0.530

*SD – Standard Deviation*

The number of students decreased as the curriculum year increased because some students (those who were admitted to FMUP after 2009) had not yet had time to complete all of the curricular years.

The mean grades of students who came from public schools were higher than the mean grades of students who came from private schools. The difference between groups blurred over the course of the curricular years. By the 6th curricular year, the difference between the groups was not significant. Of note, the variance in grades was also lower during the 6th curricular year. In fact, during this last curricular year, students usually obtained very high grades with a very low dispersion (Table 1).

Table 2 describes the proportion of students from public and private secondary schools in each curricular year with at least one failure on a CU exam.

**Table 2:** Number (n) and percentage (%) of students with at least one failure on a CU exam at medical school for each curricular year, both for students who came from public and private secondary schools

Curricular year	Private secondary school (A)		Public secondary school (B)		% Difference (A – B)	Cramer’s v	*p*-value
	N	n (%)	N	n (%)			
1	767	333 (43)	933	319 (34)	9	0.09	< 0.001
2	655	196 (30)	823	166 (24)	6	0.08	0.010
3	550	141 (26)	713	127 (18)	8	0.07	0.001
4	435	43 (10)	616	51 (8)	2	0.03	0.365
5	347	10 (3)	492	9 (2)	1	0.03	0.313
6	238	1 (0)	356	0 (0)	0	0.05	0.401

In earlier curricular years, the proportion of students from public schools with at least one failure in a CU exam was lower than the proportion of students from private schools with at least one failure of a CU exam. By the 6th curricular year, the difference between groups was almost null; however, the number of students with at least one failure of a CU exam in the last curricular year was also almost null (Table 2).

Each year the Portuguese Ministry classified some schools as those that highly inflated their internal grades. We analysed only students who were admitted to FMUP between 2009 and 2014 (the years for which we have official data about internal grade inflation). Schools that highly inflated their internal grades and had students admitted to medical s school that year (the year that grades were highly inflated) varied from 21 to 23 schools per year. There were 12 secondary schools that highly influenced internal grades during all six years of the study period (from 2009 to 2014) and had students who were admitted to FMUP. Ten of these were private and 2 public. Near half (48%) of the students admitted to FMUP from 2009 to 2014 came from secondary schools that highly inflated their internal grades that year.

Grades for 1st-year CU obtained by students who came from secondary schools that highly inflated their internal grades (both public and private schools) were on average worse than those obtained by students who came from other schools. This effect remained consistent over the six-year study period (Table 3).

**Table 3:** Mean grades for 1st-year CU based on whether students’ secondary school highly inflated their internal grades in the year of students’ admission to medical school

Year of admission to	Schools that highly inflate internal grades (A)		Other schools (B)		Mean difference (A-B)	Cohen’s d	*p*-value
Medical school	N	Mean (SD)	N	Mean (SD)			
2009	105	12.8 (1.3)	116	13.3 (1.2)	−0.5	0.40	0.002
2010	102	12.9 (1.2)	119	13.2 (1.3)	−0.3	0.24	0.089
2011	90	12.7 (0.9)	118	13.0 (1.1)	−0.3	0.30	0.049
2012	106	12.7 (1.2)	108	13.5 (1.5)	−0.8	0.60	< 0.001
2013	104	12.8 (1.1)	98	13.3 (1.3)	−0.5	0.42	0.004
2014	111	13.2 (1.2)	86	13.9 (1.3)	−0.7	0.56	< 0.001

*SD – Standard Deviation*

The percentage of students who failed at least one CU exam during the first year of medical school tended to be higher among those who came from secondary schools (both private and public schools) that highly inflated their grades than those who came from secondary schools that did not highly influence the grades. This effect was consistent across the six-year study period with the exception of 2012, during which there was no difference. However, there was also a low percentage that year (compared to previous years) of students who failed at least one CU exam during the first year of medical school. Statistical significance was only reached in the years in which there were greater differences between schools that highly inflated their internal grades and other schools, namely, 2011 and 2014 (Table 4).

**Table 4:** Number (n) and percentage (%) of students with at least one failure of a CU exam during the 1st year of medical school, both for students who came from secondary schools that highly inflated their internal grades in the year of students’ admission to medical school and those that did not

Year of admission to	Schools that highly inflate internal grades (A)		Other schools (B)		% Difference (A – B)	Cramer’s v	*p*-value
Medical school	N	n (%)	N	n (%)			
2009	105	55 (52)	116	58 (50)	2	0.02	0.724
2010	102	46 (45)	119	40 (34)	11	0.12	0.081
2011	90	32 (36)	118	26 (22)	14	0.15	0.031
2012	106	18 (17)	108	18 (17)	0	0.00	0.951
2013	104	46 (44)	98	33 (34)	10	0.11	0.124
2014	111	56 (50)	86	29 (34)	16	0.17	0.019

Linear regression results are presented in Table 5. Having attended a private secondary school (β = − 0.459, *p* < 0.001) and having attended a secondary school that highly inflated its grades (β = − 0.246, *p* = 0.003) were independent factors that significantly influenced grades during the first year of medical school (Table 5).

**Table 5:** Coefficients (simple and adjusted) from linear regression of mean UC scores from 1st year of medical school

	Simple coefficients		Adjusted^a^ coefficients	
	β	*p*	β	*p*
Secondary school type				
Public	ref		ref	
Private	−0.550	< 0.001	−0.459	< 0.001
Secondary school with high grade inflation?				
No	ref		ref	
Yes	−0.491	< 0.001	− 0.246	0.003
Student gender				
Male	ref		ref	
Female	−0.010	0.867	−0.042	0.058

^a^adjusted for type of secondary school (private vs. public), high grade inflation and student gender

The logistic regression results presented in Table 6 show that attendance of a private secondary school significantly increased the odds of students failing at least one CU exam during the 1st year of medical school (OR = 1.33, 95% confidence interval [1.02;1.75]), even after adjusting for high internal grade inflation and students’ gender (Table 6).

**Table 6:** Odds Ratios (OR, simple and adjusted) and respective 95% Confidence Intervals (95%CI) from logistic regression of whether students failed at least one CU exam during the 1st year of medical school

	Simple		Adjusted^a^	
	OR	95%CI	OR	95%CI
Secondary school type				
Public	ref		ref	
Private	1.48	[1.21; 1.80]	1.33	[1.02; 1.75]
Secondary school with high grade inflation?				
No	ref		ref	
Yes	1.50	[1.19; 1.89]	1.29	[0.98;1.69]
Student gender				
Male	ref		ref	
Female	0.88	[0.72;1.08]	1.02	[0.80;1.29]

^a^adjusted for type of secondary school (private vs. public), high grade inflation and student gender

## Discussion

Unfair access to University, particularly medical school, by students who attended private schools (with fees) prior to University has been described elsewhere in the literature, not only in Portugal [5] but also in other countries [12, 13]. Moreover, our findings were consistent with other Portuguese [14], Norwegian [15] and British studies [16], which have demonstrated that students who come from private schools perform worse in university in general and worse in medical school in particular. This evidence raises the question of whether better performance in medical school by students coming from public schools is a consequence of the fact that average students from private secondary schools are able to be admitted by taking advantage of internal grade inflation practices, whereas only highly academic students from public secondary schools, where inflation practices are used less, are able to be admitted. Our study aimed to answer this open question. Even with moderate to weak effect sizes, the present study shows that regardless of whether the secondary school of origin inflated students’ grades, medical students who come from public secondary schools performed better in medical school, at least in the early years.

Our results also show that the difference between students who come from private schools and those who come from public schools regarding their performance at medical school blurs as they advance through the curricular years. In fact, during the last curricular years of medical school, we found little differentiation in grades (students had, on average, very high grades with a very low standard deviation). Consequently, we could not determine through our study whether the difference between publicly and privately schooled students really blurs over the course of the curricular years or if this result is a consequence of less differentiation in grades in the last years of medical school. To overcome this limitation, it might be interesting in future research to compare students’ performance after the conclusion of the medical course, for example, using grades obtained from the exam used to apply for medical specialties.

Other studies have found evidence of poorer performance in university by students from private schools, but it was not clear if this is a consequence of the fact that average students from private secondary schools are able to be admitted by taking advantage of internal grade inflation practices, whereas only highly academic students from public secondary schools, where inflation practices are used less, are able to be admitted. Therefore, one strength of our results is that our study was, as far as we know, the first to show that students who came from private schools performed worse in medical school than their colleagues, even after adjusting for internal grade inflation.

Different reasons can be noted to explain the weaker performance in medical school by students who attended a private school before university. In fact, students who attended public schools before university may adapt better to medical school simply because medical school is also a public school. It is also possible that the environment and teaching methods in private secondary schools are less conducive to empowering students in independent study than those in public schools, thereby contradicting the belief that is frequently assumed to be true that students in secondary private schools are better prepared for succeeding in their academic future.

Although we studied this question in a Portuguese context, in particular, focusing on medical school, which probably employs different teaching methods than other countries, we believe that the conclusions drawn here can be generalised and applied to other countries. In fact, unfairness with regard to access to medical school and differential performance in medical school by students based on private versus public secondary education, as observed in Portugal, have also been described in other countries.

## Conclusion

Our results suggest that perhaps the admission process to medical school should be carefully studied. In fact, the scientific literature has shown that unfair access to higher education and, in particular, medical school is a concern not only in Portugal but also in other countries with different application methods. In countries such as Portugal, where secondary school grades contribute to the application score, the weight attributed to secondary school grades in the final application score should also be carefully studied.

It is the time to thoroughly debate the role of public and private secondary schools in the education process and to evaluate the emerging assumption that private schools have higher teaching standards and better prepare their students for academic success. Perhaps private schools only provide an unfair boost to medical school access.

It is important to question what can we learn from the fact that students from public secondary schools, who have an unfair disadvantage in accessing higher education, seem to be better prepared for the learning requirements and teaching methodologies of medical school than their colleagues from private schools.

## Abbreviations

CU: Course Unit
FMUP: Faculty of Medicine at the University of Porto
OR: Odds Ratio
